# Essential Role of the C-Terminal Helical Domain in Active Site Formation of Selenoprotein MsrA from *Clostridium oremlandii*

**DOI:** 10.1371/journal.pone.0117836

**Published:** 2015-02-18

**Authors:** Eun Hye Lee, Kitaik Lee, Kwang Yeon Hwang, Hwa-Young Kim

**Affiliations:** 1 Division of Biotechnology, College of Life Sciences and Biotechnology, Korea University, Seoul, Republic of Korea; 2 Department of Biochemistry and Molecular Biology, Yeungnam University College of Medicine, Daegu, Republic of Korea; Griffith University, AUSTRALIA

## Abstract

We previously determined the crystal structures of 1-Cys type selenoprotein MsrA from *Clostridium oremlandii* (*Co*MsrA). The overall structure of *Co*MsrA is unusual, consisting of two domains, the N-terminal catalytic domain and the C-terminal distinct helical domain which is absent from other known MsrA structures. Deletion of the helical domain almost completely abolishes the catalytic activity of *Co*MsrA. In this study, we determined the crystal structure of the helical domain-deleted (ΔH-domain) form of *Co*MsrA at a resolution of 1.76 Å. The monomer structure is composed of the central rolled mixed β-sheet surrounded by α-helices. However, there are significant conformational changes in the N- and C-termini and loop regions of the ΔH-domain protein relative to the catalytic domain structure of full-length *Co*MsrA. The active site structure in the ΔH-domain protein completely collapses, thereby causing loss of catalytic activity of the protein. Interestingly, dimer structures are observed in the crystal formed by N-terminus swapping between two molecules. The ΔH-domain protein primarily exists as a dimer in solution, whereas the full-length *Co*MsrA exists as a monomer. Collectively, this study provides insight into the structural basis of the essential role of the helical domain of *Co*MsrA in its catalysis.

## Introduction

Methionine sulfoxide reductases (Msrs) are ubiquitous and highly conserved enzymes that catalyze the reduction of methionine sulfoxide to methionine. Msr enzymes for the reduction of peptidyl methionine sulfoxide are classified into two protein families based on the stereospecificity of methionine sulfoxide enantiomers [1]. MsrA is specific for the *S*-isomer of methionine sulfoxide and MsrB for the *R*-isomer. Both MsrA and MsrB enzymes prevent excessive accumulation of oxidized proteins and the cytotoxic effects of reactive oxygen species [2–4]. Although MsrA and MsrB have neither homology in amino acid sequence nor similarity in structure, they share a common catalytic mechanism of the methionine sulfoxide reduction using cysteine (Cys) residues [5–7]. The general catalytic cycle of Msrs consists of three steps involving sulfenic acid chemistry. A catalytic Cys attacks the sulfoxide moiety of methionine sulfoxide to form a sulfenic acid intermediate with the concomitant release of methionine. The Cys sulfenic acid forms an intramolecular disulfide bond via interaction with a resolving Cys. The disulfide bond is then reduced by reducing agents, leading to an active form of the enzyme.

The anaerobic gram-positive bacterium *Clostridium oremlandii* contains a selenocysteine (Sec)-containing MsrA (*Co*MsrA) [8]. This selenoprotein *Co*MsrA composed of 209 amino acids contains only one catalytic Sec residue with no Cys residues and is classified as 1-Cys type MsrA. The catalytic activity of selenoprotein *Co*MsrA is 20-fold higher than that of its Sec-to-Cys version, revealing a catalytic advantage provided by selenium [8]. In addition, recycling of *Co*MsrA is not performed by the general reductant for MsrA, thioredoxin [9], and *Co*MsrA is instead efficiently reduced by glutaredoxin [9,10]. We recently determined the crystal structures of *Co*MsrA using the Cys version proteins [11]. This enzyme is found to be a structurally unusual MsrA composed of two domains, a catalytic domain and a distinct helical domain absent from other known MsrA structures [12–15]. The N-terminal catalytic domain (residues 1–144) of *Co*MsrA including the conserved active site sequence GCFWG shows a fold similar to that of other known MsrAs, which contains a central core composed of a rolled mixed β-sheet with the exterior side surrounded by α-helices. The unique C-terminal helical domain (residues 145–209) consists of five helices and interacts with the catalytic domain via the α8 helix located between the catalytic domain α1 and α5 helices. The C-terminal helical domain is conserved in some selenoprotein MsrAs [11]. Interestingly, deletion of the helical domain almost completely abolishes the catalytic activity, indicating that it is essential to *Co*MsrA catalysis [11]. Our previous structural study predicted that interaction of the helical domain with the catalytic domain might affect overall folding of the catalytic domain, maintaining active site organization [11]. However, further studies are needed to better understand the structural and functional implications of the helical domain.

In this study, the crystal structure of the C-terminal helical domain-deleted (ΔH-domain) form of *Co*MsrA was determined. The ΔH-domain structure reveals insight into the essential role of the helical domain in formation of the active site of the catalytic domain. Loss of catalytic activity of the ΔH-domain form occurs due to the active site collapse caused by loss of interaction with the helical domain and the catalytic domain. There are significant structural changes in the ΔH-domain form when compared to the full-length *Co*MsrA, including a swapped dimer structure.

## Materials and Methods

### Cloning and protein purification

A DNA sequence encoding residues 1–144 (ΔH-domain) of *Co*MsrA was PCR-amplified and cloned into expression vector pET21b as previously described [11]. The recombinant protein contained a C-terminal His-tag (LEHHHHHH). *Escherichia coli* BL21 (DE3) star cells transformed with the recombinant plasmid were grown in LB medium containing 50 μg/ml ampicillin at 37°C until the OD_600_ reached 0.5–0.6. Protein expression was induced by the addition of IPTG at a final concentration of 0.3 mM, after which cells were grown at 18°C for another 16 h. Cells were subsequently harvested by centrifugation and disrupted by sonication in ice-cold buffer A (20 mM Tris-HCl, pH 7.5, and 200 mM NaCl). Following centrifugation, the supernatant containing the amplified protein was loaded onto a HisTrap column that had been equilibrated in buffer A and the protein was eluted by a gradient increase of imidazole concentration from 20 to 500 mM. The purified protein was concentrated and applied to gel filtration chromatography on a Superdex 75 column in buffer (10 mM Tris-HCl, pH 8.0, and 100 mM NaCl). Finally, the protein purity was confirmed by SDS-PAGE analysis, and the purified protein was concentrated to 8 mg/ml.

### Crystallization and data collection

Initial crystallization of the ΔH-domain protein was carried out by the sitting drop vapor diffusion method at 20°C using Crystal Screen, Index, SaltRx, PEGRx, and PEG-ion kits (Hampton Research). Crystals were grown from a 1:1 mixture of protein solution (8 mg/ml in 10 mM Tris-HCl, pH 8.0, and 100 mM NaCl) and reservoir solution (0.1 M Tris-HCl, pH 8.5, and 3.2 M NaCl) using the hanging drop vapor diffusion method at 20°C. Prior to X-ray diffraction analysis, crystals were transferred into reservoir solution containing 20% (v/v) glycerol as a cryoprotectant and flash-frozen in the liquid nitrogen stream. The diffraction data were collected on beamline 5C at the Pohang Light Source (Pohang, Korea). All data sets were scaled and merged using the HKL2000 package [16]. The initial phases were determined by the molecular replacement method using the PHASER program [17]. The structure including residues 1–130 of the full-length *Co*MsrA (PDB ID: 4LWJ) was used as a search model [11]. Further model building was conducted using the Coot program [18], after which refinement was carried out with PHENIX [19]. The final model was analyzed and validated with MolProbity [20]. All figures were generated using PyMol (http://pymol.sourceforge.net/). Structural factors and coordinates of ΔH-domain protein have been deposited in the Protein Data Bank under accession code 4W8C. The data processing and structural refinement statistics are summarized in Table 1.

**Table 1:** Data collection and structure refinement statistics.

*Data collection*	
Space group	C222
Cell dimensions	
a, b, c (Å)	65.85, 94.47, 122.64
Resolution (Å)	50.00−1.76 (1.79−1.76)
*R*_sym_ (%)	7.2 (30.2)
*I/σ I*	49.7 (3.2)
Completeness (%)	96.4 (92.3)
Redundancy	8.9
*Refinement*	
Resolution (Å)	32.60−1.76
No. of reflections	36941
*R*_work_/*R*_free_ (%)	22.6/27.5
No. of atoms	
Protein	2190
Solvent	125
B-factors(Å^2^)	
Protein	34.05
Solvent	31.53
R.m.s. deviations	
Bond lengths (Å)	0.016
Bond angles (°)	1.613
Ramachandran plot	
Favored region	96.3
Allowed region	3.0
Outlier region	0.7

Values in parentheses are for highest-resolution shell.

### Native gel electrophoresis analysis

The purified ΔH-domain protein was diluted to a final concentration of 0.2 mg/ml with buffer (10 mM Tris-HCl, pH 8.0, and 100 mM NaCl). Reducing agent dithiothreitol (DTT) was added to the protein sample at a final concentration of 10 mM to test involvement of the disulfide bond in dimer formation. Dimer formation was also confirmed by size exclusion chromatography (SEC).

### SEC-multiangle laser light scattering

SEC coupled to multiangle laser light scattering (SEC-MALLS) was used to determine the solution molecular weight of full-length and ΔH-domain forms of *Co*MsrA. Protein samples at concentrations of 2.5 and 1.5 mg/ml for full-length and ΔH-domain forms, respectively, were applied to a WTC-015S5 column mounted on a Shimadzu HPLC system equilibrated at a flow rate of 0.5 ml/min with 20 mM Tris-HCl (pH 8.0) and 100 mM NaCl at 298K. The scattered light intensity and protein concentration of the column eluate were measured using a DAWN HELEOS II laser detector and an OPTILAB T-rEX refractive index detector (Wyatt Technology), respectively. Data analysis from both detectors was carried by ASTRA software version 6.1 (Wyatt Technology).

## Results and Discussion

### Overall structure of ΔH-domain form of *Co*MsrA

**Fig 1:**
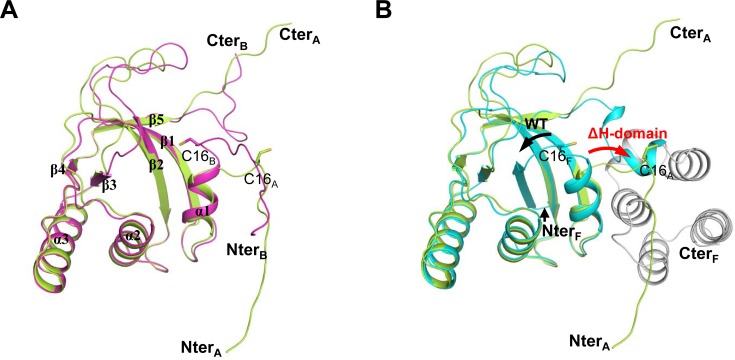
Monomer structures of ΔH-domain protein. (A) Two monomer structures of ΔH-domain protein, chains A (green) and B (magenta), are superimposed. (B) The monomer structure of chain A (green) is superimposed against the full-length *Co*MsrA, indicated by a letter F (the catalytic domain is cyan and the helical domain is grey). The directions of the N-termini of full length *Co*MsrA and ΔH-domain proteins are indicated by black and red arrows, respectively. The catalytic Cys16 residues are displayed in stick models on the overall cartoon structures.

The crystal structure of the ΔH-domain form comprising residues 1–144, corresponding to the catalytic domain, was determined at a resolution of 1.76 Å. In an asymmetric unit, there are two molecules of ΔH-domain protein, but each molecule shows different crystal structures. Refinement of crystal structure was tricky because while the structure of a molecule (chain B) of the two ΔH-domain proteins was easily solved, the other molecule (chain A) was difficult to refine. The correct phase of chain A was determined by molecular replacement using the chain B structure followed by iterative refinements. The final refined model contains chain A comprising 134 residues (7–140) and chain B comprising 137 residues (6–142). Superposition of chain A with chain B provides a root mean square deviation (r.m.s.d.) of 0.54 Å for 90 Cα atom pairs (Fig. 1A). Each chain is composed of three α-helices, five β-strands, and long loop regions. The two chains are well superimposed with respect to the α-helices and β-strand regions, but show large differences in the loop regions. In particular, both the N- and C-terminal regions show completely different conformations between these two chains. The N-terminus of chain B including residues 6–18 is extended outward in a different direction from that of chain A, while the C-terminus of chain B including residues 133–142 shows a flexible loop structure with a different conformation from that of chain A.

**Fig 2:**
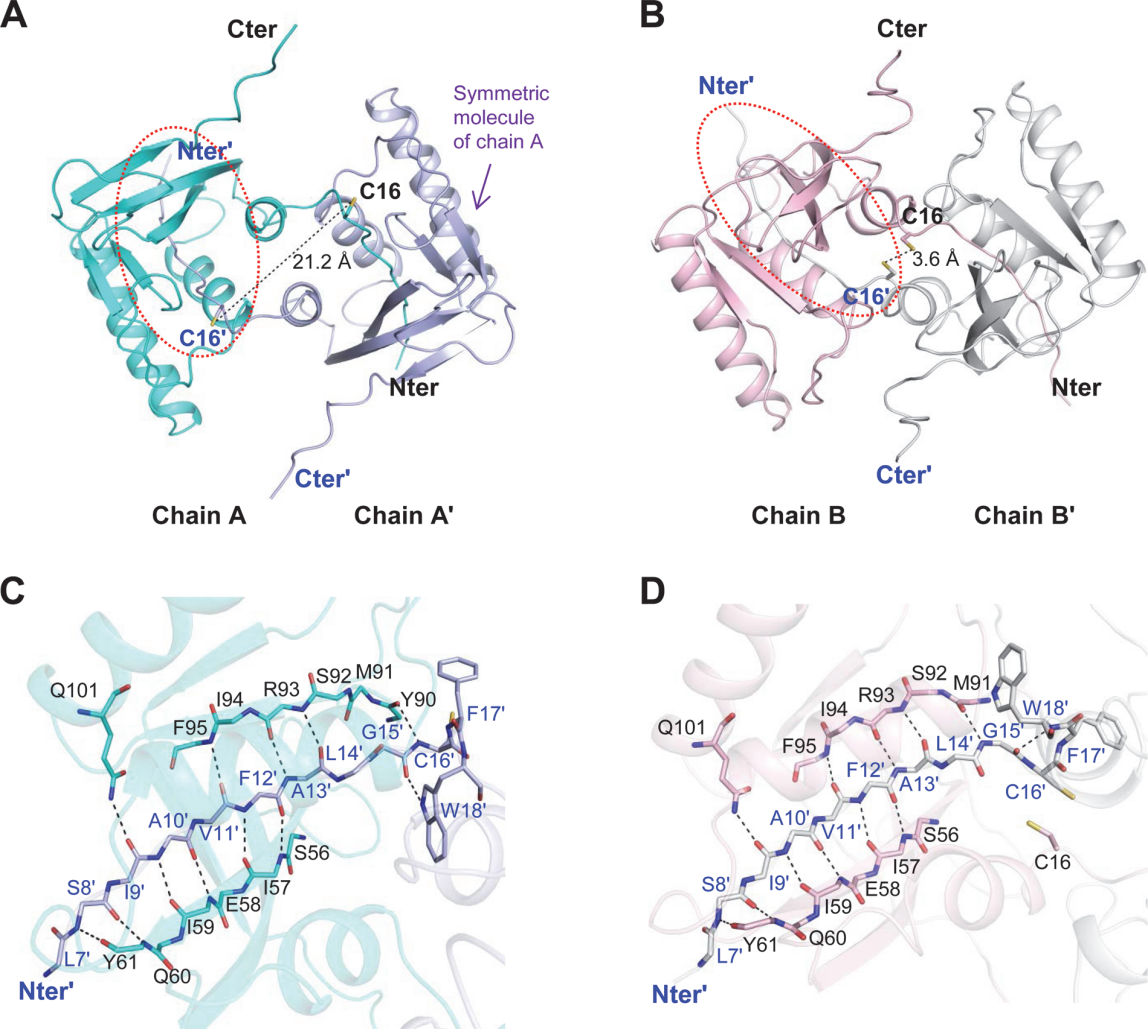
Swapped dimer formation of ΔH-domain protein. The structures of dimers A and B are displayed in (A) and (B), respectively. Each chain forms a dimer structure with its symmetric molecule. The two Cys16 residues are displayed in stick models and the distances of the two sulfur atoms are indicated by dashed lines. The dimer interaction site is indicated by a red dash lined circle in (A) and (B). The dimeric interface is magnified in (C) and (D). (C) shows the intermolecular interaction of chains A and A' and (D) shows that of chains B and B'. The dashed lines in (C) and (D) indicate the hydrogen bonds in dimer interactions. The interacting residues are represented in the backbone structure, except for residues C16, F17, W18 and Q101, which are shown with side chains. The residues of the symmetric molecule are labeled with blue letters and the "prime" sign.

As mentioned above, the ΔH-domain form comprising residues 1–144 corresponds to the catalytic domain of full-length *Co*MsrA. The catalytic domain structure of full-length *Co*MsrA consists of five α-helices and six β-strands, which fold into a rolled mixed β-sheet surrounded by α-helices [11]. Notably, the ΔH-domain form contains three α-helices and five β-strands. Superimposition of two chains of the ΔH-domain form to the full-length *Co*MsrA gives r.m.s.d. values 0.62 Å for 97 Cα and 0.59 Å for 98 Cα atom pairs for chains A and B, respectively (Fig. 1B). The monomer structure of the ΔH-domain protein folds into a similar rolled mixed β-sheet with three helices located at the exterior sides. However, the N- and C-terminal structures of ΔH-domain protein differ from the catalytic domain of the full-length *Co*MsrA form. The N-terminal region of full-length *Co*MsrA comprising residues 6–18, which includes the catalytic Cys16, forms a β-strand (β1) that participates in the rolled mixed β-sheet. In contrast, the N-terminal end migrates in the opposite direction in the ΔH-domain form, where it interacts with another symmetric molecule in the crystal (Fig. 2). This N-terminal region is inserted in between two β-strands (β2 and β3) of the symmetric molecule and participates in the rolled mixed β-sheet in a swapped dimer (discussed in detail below). The C-terminal region of the ΔH-domain protein comprising residues 133–142 does not form any helices near the central β-sheet, while the corresponding region of the full-length form participates in formation of two short helices [11].

### Swapped dimer structures of ΔH-domain protein

The ΔH-domain protein forms two different dimer structures in the crystal. Two chains, A and B, form individual dimer structures with their symmetric molecules: dimer A, chains A and A'; dimer B, chains B and B' (the “prime” sign indicates symmetric molecules; Fig. 2A and B). The dimers are made by swapping of the N-terminus between two molecules. Each N-terminus including residues 6–18 extends outwards from the original molecule of full-length form, intruding between β2 and β3 of another symmetric molecule and forming a complete rolled mixed β-sheet. The intruded N-terminus interacts with both β-strands in anti-parallel orientation via multiple hydrogen bonds (Fig. 2C and D). The Nε2 atom of Gln101 placed on the nearby α3 helix also forms a hydrogen bond with the carbonyl oxygen atom of Ile9'.

**Fig 3:**
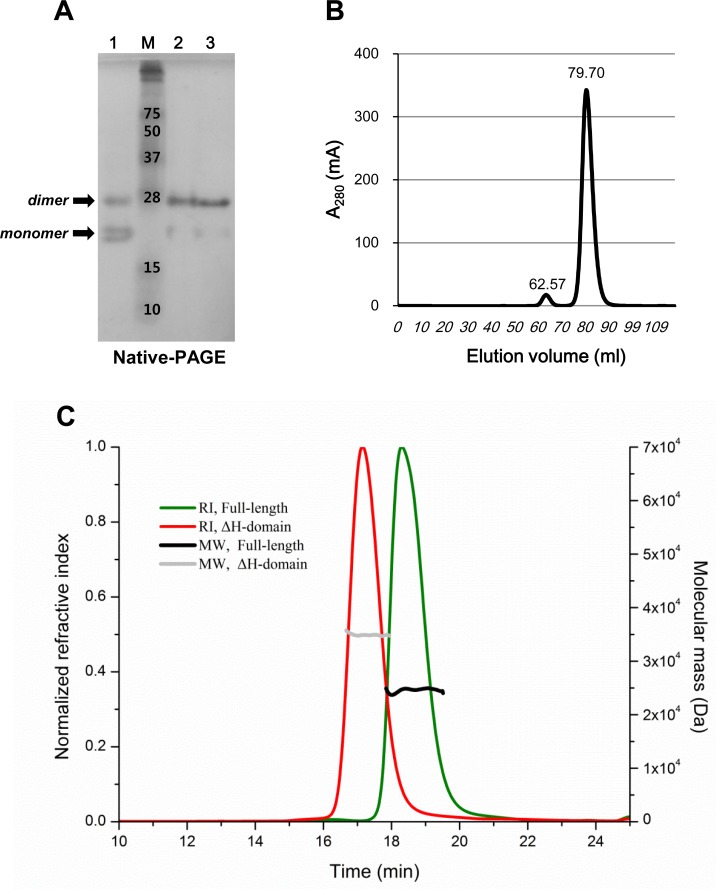
Dimer formation of ΔH-domain protein in solution. (A) The purified ΔH-domain protein (0.2 mg/ml) was loaded onto native-PAGE. M, protein marker; lane 1, ΔH-domain protein with 2% SDS; lane 2, ΔH-domain protein only; lane 3, ΔH-domain protein with 10 mM DTT. (B) The gel filtration profile of ΔH-domain protein at the final purification step is shown. Two peaks were observed upon analysis with a Superdex S75 16/60 column (GE Healthcare). The elution volume is indicated on the top of each peak. The elution volume of 79.70 ml corresponds to a dimer size (30 kDa) and 62.57 ml to a tetramer size (60 kDa). (C) SEC-MALLS analysis of ΔH-domain and full-length forms of *Co*MsrA. The refractive index (RI) of the column eluate is plotted as a function of time. The weight-averaged molecular weight (MW) of the material in the eluate is calculated from light-scattering measurements. The calculated molecular masses are 35,010 Da for the ΔH-domain protein and 24,570 Da for the full-length protein.

The oligomeric state of the ΔH-domain protein in solution was analyzed by native-PAGE and gel filtration (Fig. 3A and B). The ΔH-domain protein predominantly existed as a dimer in solution, which is in agreement with the crystallographic data. Notably, the full-length *Co*MsrA is a monomeric protein in solution and in crystal [11]. Native gel analysis showed that the dimeric ΔH-domain cannot be dissociated by the addition of the reducing agent, DTT, indicating no involvement of the disulfide bond between Cys16 residues. In addition, SEC-MALLS analysis was performed to determine the solution molecular masses of ΔH-domain and full-length forms of *Co*MsrA (Fig. 3C). The calculated molecular masses are 35,010 Da for the ΔH-domain protein and 24,570 Da for the full-length protein. These results support that the ΔH-domain protein exists as a dimer in solution, whereas the full-length protein exists as a monomer.

The ΔH-domain form of *Co*MsrA is almost inactive even in the present of DTT as reductant. In contrast, a truncated form of *E. coli* MsrA (containing residues 42–194), which corresponds well to the ΔH-domain form of *Co*MsrA, shows an activity comparable to the full-length wild-type in the presence of DTT [21]. The *E. coli* MsrA truncated protein is present as a monomer in solution as the full-length wild-type [21]. The enzymatic and oligomeric properties of the *Co*MsrA ΔH-domain form are quite different from those of the *E. coli* MsrA truncated form.

The two dimer structures have similar N-terminus swapping, but different interface areas. The buried areas are 2186 Å^2^ for dimer A and 2607 Å^2^ for dimer B, which correspond to 13% and 16% of the surface areas, respectively. Dimer B is more compact than dimer A by the PISA analysis [22]. The two dimers also show different structural features near the Cys16 residue, which is the catalytic residue of *Co*MsrA and the only Cys in the entire protein sequence [8]. In dimer A, Cys16 residues of the two molecules are located far away from each other at a distance of 21.2 Å between two sulfur atoms. In contrast, in dimer B, Cys16 residues are closely located at a distance of 3.6 Å, but no disulfide bond is observed. The two chains in the asymmetric unit (A and B) show different conformations in the loop regions, including N- and C- termini (Fig. 1A). The N-terminus is strongly associated with the dimer formation, whereas the C-terminus plays no role in dimer formation (Fig. 2A and B). Therefore, the conformation of the N-terminus is critical to formation of the dimer structure and eventually affects the conformations of the two dimer structures. The N-terminus from Leu7' to Trp18' participates in interactions between anti-parallel β-strands (Fig. 2C and D), and these interactions determine the different conformations of the swapped dimers. The residues Leu7'–Leu14' participate in the dimer formation by making multiple hydrogen bonds with the symmetric chain, whereas residues Gly15'–Trp18' are involved in determination of the dimer conformation. Specifically, these residues form hydrogen bonds in different orientations between the two dimer structures. In dimer A, the amide nitrogen and carbonyl oxygen atoms of Cys16' form hydrogen bonds with the carbonyl oxygen atom of Tyr90 and the amide nitrogen atom of Trp18', respectively. The Nε1 atom of the indole ring of Trp18' forms a hydrogen bond with the carbonyl oxygen atom of Gly15'. In dimer B, a hydrogen bond is observed between the carbonyl oxygen atom of Gly15' and the amide nitrogen atom of Trp18'. This different arrangement of hydrogen bonds leads to different dimer structures (Fig. 2C and D).

### Active site collapse in ΔH-domain protein

**Fig 4:**
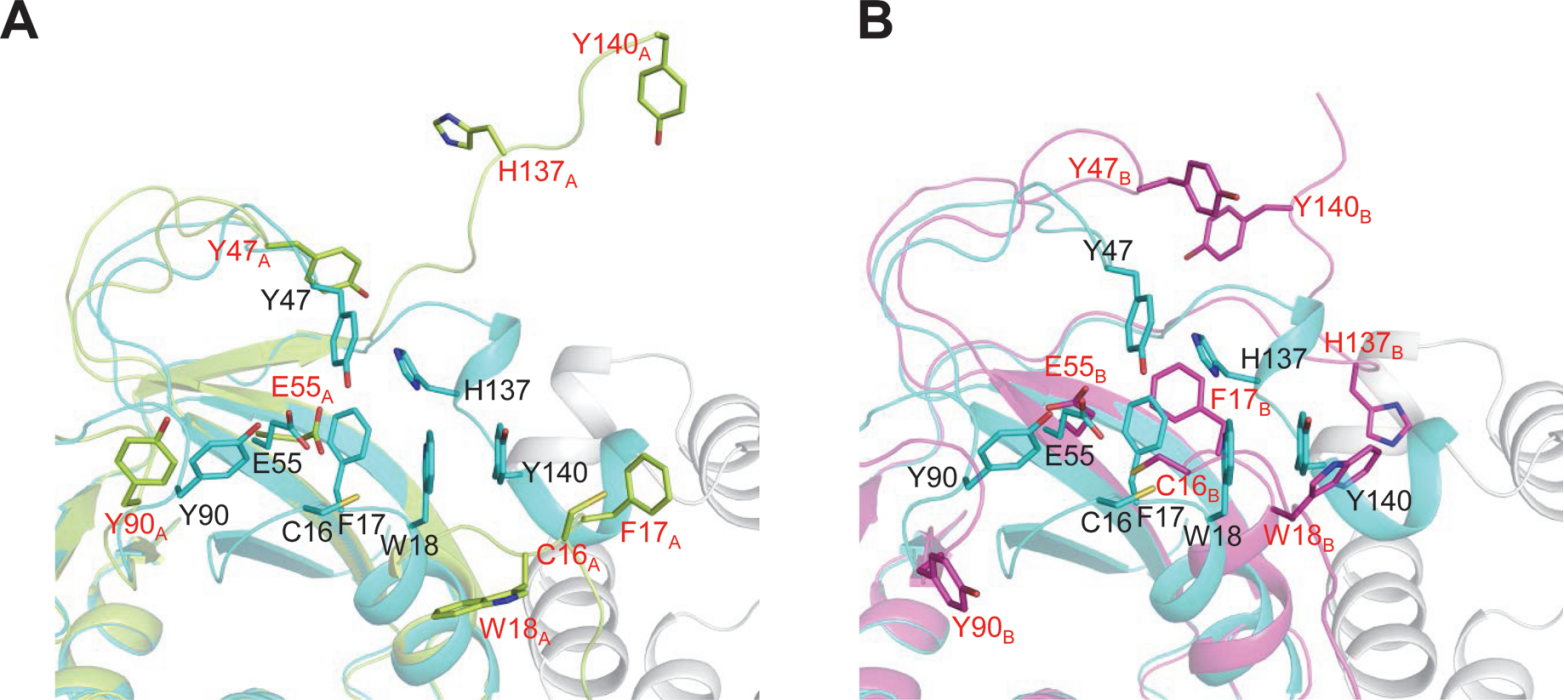
Active site comparison of ΔH-domain and full-length *Co*MsrA proteins. (A) Superposition of the active sites of ΔH-domain chain A and full-length *Co*MsrA (chain A, green; *Co*MsrA, cyan for the catalytic domain and grey for the helical domain). (B) Superposition of the active sites of ΔH-domain chain B and full-length *Co*MsrA (chain B, magenta). The active site residues are represented in stick models. The residues of chain A and chain B are labeled with red letters and those of *Co*MsrA with black letters.

The catalytic domain of *Co*MsrA consisting of residues 1–144 corresponds well to other known MsrAs in sequence and structure [11]. Nevertheless, the ΔH-domain protein shows almost no activity [11]. Our main interest in this study was to determine why the distinct helical domain is crucial to the catalytic activity of *Co*MsrA protein. The helical domain has several hydrogen bond interactions via the α8 helix with the two neighboring helices (α1 and α5) of the catalytic domain [11]. However, there are no direct interactions with the active site residues of the catalytic domain. In a previous study, we predicted that these tight interactions between the catalytic and helical domains may be important for holding the active site structure of the *Co*MsrA catalytic domain [11]. The active site of *Co*MsrA is similar to other MsrAs, including *E. coli* and bovine enzymes. The active site includes the highly conserved ^15^GCFWG^19^ motif and residues Tyr47, Glu55, Gln89, Tyr90, His137, and Tyr140 (Fig. 4A and B) [11]. For the catalytic activity, these active site residues must be correctly organized and located to form a functional active site. The active site is composed of two opposite characterized parts, a hydrophilic portion needed to accommodate the sulfoxide oxygen of substrate and a hydrophobic part for the ε-methyl group. Residues Tyr47, Glu55, and Tyr90 form the hydrophilic part, while residues Phe17, Trp18, His137, and Tyr140 form the hydrophobic part (Fig. 4A and B). These active site residues show a completely different orientation in the ΔH-domain protein compromising seriously the active site. Changes in residue orientation in the ΔH-domain protein can be divided into minor, local, and major alterations. The Glu55 residue located on the central β-sheet shows a minor alteration without changes in the Cα backbone. Residues Tyr47 and Tyr90 located on the two loop regions show a local alteration with changes in the Cα backbone following movement of the loop regions. Residues Cys16–Trp18, His137 and Tyr140 forming the hydrophobic region show major alterations on the N- and C-termini of the ΔH-domain protein. These two termini show remarkable structural changes caused by deletion of the helical domain. The residues on these termini differ greatly from their active site orientation. Loss of interactions with the catalytic domain by deleting the helical domain leads to collapse of the active site, thereby causing a loss of activity. Collectively, these data suggest that the helical domain is essential to maintenance of active site organization of the catalytic domain of *Co*MsrA, as previously predicted.

### Possible determinants of domain swapping

Domain swapping is a mechanism of protein oligomerization in which the structural elements (or domains) of individual monomers are interchanged between identical partners [23]. The observation that only the ΔH-domain protein dimerizes by swapping the N-terminal strand leads to questions about the topology or sequence elements that induce the swapping mechanism. The C-terminal region, including Tyr140, of the ΔH-domain protein is transformed into a flexible loop from the original helices structure. This transformation destroys the π-π stacking interaction between Tyr140 residue of the C-terminus and Trp18 residue of GCFWG motif. The loss of this π-π interaction would make the GCFWG motif more flexible. The flexible GCFWG motif would act as a hinge loop to induce the N-terminus swapping [23]. Based on the scoring of amino acid propensity for the hinge region [24], Cys16, Phe17, and Trp18 residues of the GCFWG motif retain a high propensity for the hinge region. The Trp18 residue shows a large conformational change even between the two domain-swapped dimer structures (Fig. 2C and D).

## Conclusions

The crystal structure of *Co*MsrA lacking the C-terminal helical domain is described. The active site is completely destroyed in the ΔH-domain protein, demonstrating its loss of catalytic activity. In addition, the ΔH-domain protein forms a dimer structure via swapping of the N-terminus and is predominantly present as a dimer in solution, whereas the full-length *Co*MsrA is a monomeric protein.
